# Legislative Interference in Medical Institutions

**Published:** 1856-04

**Authors:** 


### LEGISLATIVE INTERFERENCE IN MEDICAL INSTITUTIONS.

"It is a great evil, that Legislatures are ever ready to grant charters to all grades of Colleges, whenever application is made for the same, without stopping to consider the circumstances. It is too often the case, that medical men undertake to get up a college for their own personal ambition or promotion; and to the injury of the profession. Unless the institution can command the best talents, it is not worthy of patronage."

The above is an extract of a letter from Dr. Coons, of St. Louis, to the American Medical Gazette, written from Richmond, Virginia, where it appears he was sojourning at the time.

The looseness on the part of legislators and others in power, in granting charters and thus encouraging the unnecessary multiplication of schools of medicine, is indeed an evil which has done much to injure the cause of medical education. If colleges would be supplied when the wants of certain localities demanded their erection, all would be well. But one may supply the wants of a district and succeed well, but another projected might disturb its healthfulness. The second may be projected by just such persons as are referred to in the above extract, and actuated by such motives, manage to enter upon a plan by which it may be destroyed and have their ambition gratified by raising, Phœnix like, another from its ashes. Our brethren in the State well know that two attempts of this kind have been unsuccessfully made within a few years, to break the State Institution down. Last summer it was heralded forth with a flourish of trumpets and staring letters that *one grand transcendental* and *unrivalled institution* was to be opened in this city, in which, and in *that only*, would medical science be taught and medical truths promulged to an *astounded* profession.

As reference has already been made to this notable enterprise, the motive explained, and the history given, with more attention than it really deserved, had it not been necessary to disabuse the public mind, we shall not, therefore, allude to it now, except to say that such Theopolemeon spirits as the leaders of that may, if they would, be taught a lesson from past experience.

It is a source of bitter mortification to medical men, and the complaint is universal, that non-medical men do not, or will not, comprehend nor understand the true interests of medical science. And this is the more strange, and yet not the less mortifying, when all know well that they expect such proficiency at their hands. The physician must be well qualified, because so much is expected of him; and yet the intelligent members of community actually do less to sustain medical schools than any other branch of learning or science.

In this State an effort was made by an officer high in authority, appointed by the people to protect, foster, and encourage our educational interests, and yet that officer, with this sacred trust in his hands, with an obligation no less solemn than an oath, to discharge faithfully the trust given him by the people, lent himself to certain influences and used his official bias against this Institution, the only department of the University, then or now, in a state of prosperity and success, with the view to its downfall and destruction. But the profession and the people, had too much intelligence and independence, and too much regard for institutions calculated to contribute to the well-being of society, to listen to his *dicta*, or be guided by his views or opinions. The profession looked upon it as but the gratification of a hostility which he doubtless indulged in toward the regular profession, as he himself was not only a believer in, but actually practiced one of the heterodox *isms* of the day.

We once knew a member of the legislature, who having been sick, arose from his couch and declared that his "*doctor had saved his life*," and yet notwithstanding this extravagant expression, he took his seat and in a few days voted "*Aye*" on a bill to establish a "*Steamsonian Tom-doctor*" manufactory. We looked at him with astonishment, but with a larger share of contempt for his inconsistency and ingratitude, and sincerely thought the *game* not worth the exercise of the doctor's *skill* or his *powders*.

Lawyers despise "pettifoggers," and yet they get into the legislature in swarms, and are often found following the light of some *will-o-the-wisp* in medicine.

The clergy too are foremost in the building up of institutions of learning, and claim for those who enter the ministry, the highest and most liberal acquirements. And yet after all, their names are often found endorsing some vile nostrum and their reverend smiles are as often found bestowed upon some charlatan or notorious quack, and his pious example is followed by a majority of his flock. Of course his precepts would not include words of encouragement to sustain regular medical schools. Legislators enact sanitary laws for the protection and preservation of the public health, to punish offenders for permitting filthy nuisances to exist, to provide for quarantines and hospitals, and yet they will permit, and often actually encourage, the establishment of *sinks*, from which flow *filth*, giving rise to poisonous *effluvia* most *rank* and *active* in the production of more mischief among the people than all the stagnant pools, decomposing vegetable, or putrid animal matters combined. And yet these *sinks* send forth their vicegerents of destruction, are encouraged and sustained by *wise* legislators, by men of influence, by the people at large; as if no danger lurked in their way,—as if no stagnant pool was not then poisoning them,—as if no putrid carcass was not busily throwing its stench in their very nostrils. The secular journals of the day are daily giving *pabulum* to quackery, and upon the very same page perhaps, there is found the bold assumption that they are the "*sentinels upon the watch tower, guarding the people!*"

It is through, and by such unholy influences as these that establishments for the manufacture of instruments of the most approved models, warranted "to kill" at the shortest notice, are erected, sustained, and encouraged.

The principle of public safety would be startled into alarm, if in the midst of a densely populated city, an establishment would be founded in which death-dealing instruments were manufactured, and intended for use by the first infuriated mob that might rise in a mad rebellion against the peace and safety of the citizens. They would rise *en masse*, and the most emphatic denunciations would find universal utterance. The majesty of the law would be appealed to and the offenders arrested and punished for their treasonable purposes and designs. There are other "*infernal machines*" than that of "Arrison's," but with this difference, the one was smuggled where it could do its errand of death, the others exist by *permission* of law and protected by its authority. How sickening to contemplate such blind indifference, apathy, and stupidity.

Under such circumstances, where can the profession look for "aid and comfort," except in their own inherent powers, and in united efforts for their own defence? What incentive have they to labor and plod, to seek and to search, and thus drudgingly drag through a career of usefulness, all the while discouraged at the public apathy manifested for their efforts; to see the low, ignorant empiric often placed superior to him, and that too by persons claiming intelligence? All the powerful influences arrayed against them, legislative, judicial, religious, moral, and benevolent, or if not actually hostile, at least, most witheringly cold and apathetic. We repeat, where can they look for a smile of encouragement, save in the few enlightened, upright, and appreciating members of society.

Behold the outrage which has been perpetrated upon the profession, of common intelligence, and the Faculty of the University of Michigan. The Regency are struggling against the exercise of a mandate, which for injustice and outlawry, is without precedent in the history of this, or any other country. The Turks, a people despised because in Mahomedan darkness, would not perpetrate so monstrous a wrong, for they have recently decreed that empiricism should not find an abode among them. What a profound and erudite legislature that body of men must have been who enacted that law which would be condemned by the heathen? The history of science in this country will place this remarkable instance upon record, in order to perpetuate the glory of that State as one of the most sublime exhibitions of the spirit of progress. Bright and brilliant will be that spot, to which the lovers of science, in all coming time, may look back with exultation, and indulge in thoughts of veneration for the memory of those exalted spirits who had placed it upon its pages. "Old Scotia" exulted in its national valor, which had descended in a long line of ancestry from the ancient fathers of the realm, but it was reserved for the "Wolverines" to write a still more brilliant history. If the "regency" would but bestow upon those who projected so transcendant an idea, and those who advocated the measure, a dignitary title as a "dukedom," or something else at the disposal of their *royal* power, the title might descend down a line of illustrious descent, marked by certain peculiar physical attributes, to which we shall allude presently. There must be a sublimity in their *littleness* peculiarly awe-inspiring.

"Oh homœpathy, homœpathy oh!!"

As Pisistratus has perpetuated the memory of old Homer, whose harp strings which still quiver with sweetest melody, through his agency, and which so powerfully seize upon, and has ruled the mind down to the present time, so may some one in future time, keep alive the memory of the "great Lilliputian," who may happen to be the appointee, and preserve faithfully the virtues of so illustrious a personage. Greece, yea classic Greece, the great "school marm" of the world, had her Athenian University. She again had *her* Forum, *her* Lyceum, and *her* Porch, but it was reserved for the nineteenth century to improve upon the organization of a University, and the glory to belong to "Michigan," which has added a department of "*infinitesimalism.*" A happy spirit of transcendental inspiration came soothingly over the feelings of the distinguished statesmen of that State, who yielding to the dulcet influences, the mighty scheme was conceived and finally born. Literary, law, and medical departments were not sufficient to satisfy their love of glory, but there must be superadded a climax which was to place the institution in the highest niche among the institutions of the world. The University of Greece could boast of a Zeno, the learned Plato, and the philosophic Aristotle, but it remains to be seen whom the regency will be compelled to place in the new position —some greater doubtless, than an Aristotle—some Georgias Leontinus, we suppose, who can be fluent and eloquent when there is "*nothing*" to discuss and *no one* to speak to.

If the same wise legislatures again assemble, the next chair provided for will be "Free Love," and after this again "*spiritualism*" in particular, and *necromancy* in general. To the Faculty we send greeting, if they are disposed to feel and act as neighbors, our sympathies; and to the prefession of the State we appeal in behalf of its dignity, to aid in resisting this foul encroachment, and the deflowering of the honor of medical science in their State. The regency would do well to show, among the other things they are required to exhibit, the class of animal organization to which, from certain *aural* developments, those who have aimed to coerce them into the perpetration of a monstrous wrong, legitimately and properly belong.

If such corrupt powers prevail against them, then indeed did the members of the American Medical Association select a most unfortunate place for their assemblage—among those who have dishonored the profession, by insulting its dignity and honor. And yet they can beard the "lion in its den," and offer sympathies to our friends in that State, and bid them hope for better things. At the same time it can say to the enemies of medical science in that State, that if they desire to crush the University, they cannot more effectually do it than by persisting in their present course of madness and wrong.

But medical science has within itself its own protecting ægis. It will stand alone if let alone. It is often more injured by legislation, than benefitted, even when the motive has been good. The history of legislation in this country will show that the profession of medicine has been neglected, and not neglected only, but laws, decisions, and legislation has been ever against it. The profession must stand by and defend its own rights, its dignity and honor.

